# Specific breast cancer prognosis‐subtype distinctions based on DNA methylation patterns

**DOI:** 10.1002/1878-0261.12309

**Published:** 2018-05-21

**Authors:** Shumei Zhang, Yihan Wang, Yue Gu, Jiang Zhu, Ce Ci, Zhongfu Guo, Chuangeng Chen, Yanjun Wei, Wenhua Lv, Hongbo Liu, Dongwei Zhang, Yan Zhang

**Affiliations:** ^1^ College of Bioinformatics Science and Technology Harbin Medical University China; ^2^ Department of General Surgery The Second Affiliated Hospital of Harbin Medical University China

**Keywords:** breast cancer, consensus clustering, DNA methylation, molecular subtypes

## Abstract

Tumour heterogeneity is an obstacle to effective breast cancer diagnosis and therapy. DNA methylation is an important regulator of gene expression, thus characterizing tumour heterogeneity by epigenetic features can be clinically informative. In this study, we explored specific prognosis‐subtypes based on DNA methylation status using 669 breast cancers from the TCGA database. Nine subgroups were distinguished by consensus clustering using 3869 CpGs that significantly influenced survival. The specific DNA methylation patterns were reflected by different races, ages, tumour stages, receptor status, histological types, metastasis status and prognosis. Compared with the PAM50 subtypes, which use gene expression clustering, DNA methylation subtypes were more elaborate and classified the Basal‐like subtype into two different prognosis‐subgroups. Additionally, 1252 CpGs (corresponding to 888 genes) were identified as specific hyper/hypomethylation sites for each specific subgroup. Finally, a prognosis model based on Bayesian network classification was constructed and used to classify the test set into DNA methylation subgroups, which corresponded to the classification results of the train set. These specific classifications by DNA methylation can explain the heterogeneity of previous molecular subgroups in breast cancer and will help in the development of personalized treatments for the new specific subtypes.

**Abbreviations**
AUC: area under curve
CDF: consensus cumulative distribution function
CpG: cytosine preceding a guanosine
CTL: cytotoxic T lymphocytes
CV: coefficient of variation
DMRs: differentially methylated regions
GEO: Gene Expression Omnibus
knn: k‐nearest neighbours
QDMRs: quantitative differentially methylated regions
ROC: receiver operating characteristic curve
RSEM: RNA‐Seq by expectation‐maximization
SD: standard deviation
TCGA: The Cancer Genome Atlas

## 1 Introduction

Breast cancer is a highly complex and heterogeneous disease with different molecular profiles, clinical responses to therapeutic agents and prognoses (Perou *et al*., 2000). Tumour heterogeneity has led to the various subtypes of breast cancer, which have different sensitivities to chemotherapy and prognoses (Tazaki *et al*., 2013), highlighting the importance of precision/personalized medicine. Precision medicine aims to optimize the effectiveness of disease prevention and treatment by tailoring personalized treatments based on an individual's specific genetic lesions, biomarkers, environment and lifestyle (Reitz, 2016). Precision medicine has been applied in clinical practice since the initial efforts to classify disease subtypes and administer specific treatments based on diagnoses. Precision medicine has been studied in a variety of diseases including Alzheimer's disease (Reitz, 2016), Parkinson's disease (Bu *et al*., 2016) and cancer (Arnedos *et al*., 2015; Prados *et al*., 2015).

Decades ago, molecular differences in breast cancers were studied to improve treatments and make up for the lack of histological types ofeast breast cancer. Microarray‐based gene expression profiling of breast tumours identified at least five major intrinsic subtypes: Basal‐like, Luminal A, Luminal B, Human epidermal growth factor receptor 2‐positive/estrogen receptor‐negative (HER2^+^/ER^−^) and Normal Breast‐like (Prat and Perou, 2011). Currently, many research institutions are dedicated to studying breast cancer molecular subtypes. Chen *et al*. (2015) studied 187 young breast cancer patients (< 40 years old) by univariate and multivariate analyses and proved, consistent with previous studies, that molecular subtypes are independent prognostic factors for young breast cancer patients, and that triple negative breast cancer has the highest risk of recurrence and death. Using hierarchical clustering, Sorlie *et al*. (2001) identified at least two subgroups of ER^+^ breast tumours that showed differences in gene expression profiles and prognosis. In addition, they identified a luminal subgroup that had better prognosis. Curtis *et al*. (2012) classified breast tumours into 10 IntClust subclasses by combining gene expression and copy number data from 2000 breast tumours.

Although genetic alterations such as mutations, rearrangements and copy number changes are known to influence carcinogenesis, epigenetic alterations including DNA methylation also play an important role in cancer development. DNA methylation occurs when DNA is modified by the addition of a methyl group to the 5′ position of a cytosine preceding a guanosine (CpG). CpGs are often found at high densities in ‘CpG islands’, particularly within the promoter regions of genes. Hypermethylation of CpG islands can result in the transcriptional silencing of tumour suppressor genes in cancer, whereas CpG hypomethylation may lead to oncogene activation (Antequera and Bird, 1993). Fleischer *et al*. demonstrated that epigenetic changes are present in ductal carcinoma *in situ* (DCIS), the earliest stage of breast cancer progression, providing evidence for the possible use of DNA methylation‐based markers in the clinic and highlighting the importance of epigenetic changes in cancer (Fleischer *et al*., 2014). Moreover, DNA methylation profiling beyond promoters could be a potential clinical tool for characterizing the tumour microenvironment and cell typing within tumours, including breast cancer (Jeschke *et al*., 2015). This assay also has the potential to evaluate tumour immune responses and improve the diagnosis and treatment of breast and other cancers (Jeschke *et al*., 2017). A previous study showed that BRCA1 promoter methylation was correlated with clinical breast cancer stages (Chen *et al*., 2009). Thus, DNA methylation status can be used as a marker for breast cancer molecular subtyping. In 2017, Thomas *et al*. presented a DNA methylation signature (SAM40) that includes 41 significantly differentially methylated genes, and showed that it could segregate Luminal A patients into two subgroups: a good prognosis group and a poor prognosis group (Fleischer *et al*., 2017). Ronneberg *et al*. (2011) identified three major clusterings of breast tumours based on methylation profiles, one primarily consisting of tumours of myoepithelial origin and two comprising tumours of mainly luminal epithelial origin. Holm *et al*. (2010) found that the Basal‐like, Luminal A and Luminal B breast cancer molecular subtypes harbour specific methylation profiles by analysing the methylation status of 807 cancer‐related genes in 189 fresh frozen primary breast tumours and four normal breast tissue samples using an array‐based methylation assay. Furthermore, they also integrated different types of genome‐wide data, not limited to methylation, to improve the characterization of breast cancers (Holm *et al*., 2016). Conway *et al*. (2014) obtained four DNA methylation subtypes of breast cancer by DNA methylation profiling; however, their classification may not be detailed enough, and the specific sites associated with each category are unclear.

In this study, we addressed breast tumour classification by identifying specific prognosis‐subtypes based on DNA methylation profiles of breast cancer from The Cancer Genome Atlas (TCGA) database (Tomczak *et al*., 2015). This classification system may help find new breast cancer markers or molecular subtypes to more accurately subdivide breast cancer patients. Furthermore, our classification system provides guidance for clinicians regarding diagnoses and personalized treatments by identifying differences in prognoses for each epigenetic subtype. Additionally, our criteria will provide more targets for breast cancer precision medicine by finding specific molecular markers for each subtype.

## 2 Materials and methods

### 2.1 Data pre‐processing and the initial screening of DNA methylation loci in breast cancer

Breast cancer DNA methylation data generated with the Illumina Infinium HumanMethylation450 BeadChip array were downloaded from the TCGA data portal (Cancer Genome Atlas Research *et al*., 2013). The methylation level of each probe was represented by the β‐value, which ranges from 0 to 1, corresponding to unmethylated and fully methylated, respectively. Probes with missing data in more than 70% of the samples were removed. The remaining probes with not available (NAs) were imputed using the k‐nearest neighbours (knn) imputation procedure. The ComBat algorithm in sva R package (Leek *et al*., 2012) was used to remove batch effects by integrating all the DNA methylation array data and incorporating batch and patient ID information. Unstable genomic sites, including CpGs in sex chromosomes and single nucleotide polymorphisms, were removed. Because DNA methylation in promoter regions strongly influences gene expression, we selected CpGs in promotor regions. Promoter regions were defined as 2 kb upstream to 0.5 kb downstream from transcription start sites. Finally, we selected samples that had gene expression profiles. In total, 669 breast tumours were used for the analysis.

Next, we separated the dataset into two cohorts: a train set and a test set. The criteria for these grouping were as follows: (a) the samples were randomly divided into two groups; (b) age distribution, staging, follow‐up time and death ratio were similar in the two groups. Clinical information was missing for 11 samples; these samples were randomly assigned to the train and test sets.

### 2.2 Data pre‐processing of the breast cancer expression dataset

RNA‐SeqV2 level 3 expression data, quantified as RNA‐Seq by expectation‐maximization (RSEM) were downloaded from the TCGA database. The expression data were processed as follows: zero‐valued entries were replaced by the minimal positive value of the dataset; the expression values were logarithmically transformed (base 2) to normalize the data; and batch effects were processed using ComBat function in sva package. Finally, we selected 669 breast tumour samples with both expression profiles and DNA methylation profiles for final analysis.

### 2.3 Determining classification features by COX proportional risk regression models

The aim of this study was to obtain prognostically determinant breast cancer molecular subtypes. Thus, CpG sites that significantly influenced survival were used as classification features. First, univariate COX proportional risk regression models were constructed with methylation levels of each CpG site, age, stage, ER status and survival data of the cases. Then, the significant CpGs obtained from univariate COX proportional risk regression models were introduced into multivariate COX proportional risk regression models, using age and stage as covariates, which were also significant in the univariate models. Finally, the CpG sites that were still significant were used as classification features. COX proportional hazard models were fitted with methylation levels of CpGs using the coxph function in survival package R, with demographic and clinical attributes (age and stage) as covariates in the multivariate analysis. For each CpG *i*, the multivariate COX proportional risk regression model formula was defined as follows:

(1) $$h{\left(t,x\right)}_{i}\;\;=\;h0\left(t\right)\exp\left(\beta_{\mathrm{methy}}{\text{methy}}_{i}+\beta_{\mathrm{age}}\text{age}+\beta_{\mathrm{stage}}\text{stage}\right)$$

where methy_*i*_ was the vector of the methylation level of CpG *i* in samples, and age and stage represent the vectors of age and stage of the cases, respectively, and β_methy_, β_age_ and β_stage_ were the regression coefficients. The *P*‐values for the COX regression coefficients were adjusted using the Benjamini–Hochberg false‐discovery rate for multiple comparisons.

### 2.4 Consensus clustering to obtain molecular subtypes associated with breast cancer prognosis

Consensus clustering was performed using the ConcensusClusterPlus package in R (Wilkerson and Hayes, 2010) to determine subgroups of breast tumours based on the most variable CpG sites. The algorithm began by subsampling a proportion of items and features from the data matrix, where each subsample was partitioned into up to *k* groups by a user‐specified clustering algorithm: *k*‐means, hierarchical clustering or a custom algorithm. This process was repeated for a user‐specified number of repetitions, providing a method of representing the consensus across multiple runs of the clustering algorithm and assessing the stability of the discovered clustering. Pairwise consensus values, defined as ‘the proportion of clustering runs in which two items are grouped together’, were calculated and stored in a consensus matrix for each *k*. Then, for each *k*, a final agglomerative hierarchical consensus clustering using a distance of 1 – consensus values was completed and pruned to *k* groups, which were called consensus clustering. This algorithm determined ‘consensus’ clustering by measuring the stability of clustering results from the application of a given clustering method to random subsets of data. In each iteration, 80% of the tumours were sampled, and the *k*‐means algorithm, with the Euclidean squared distance metric was used:

(2) $$d=\sqrt{\sum_{k=1}^{N}{\left(x_{k}-y_{k}\right)}^{2}}$$

with *k* = 2 to *k* = 20 groups; these results were compiled over 100 iterations. After executing ConsensusClusterPlus, we obtained the cluster consensus and item‐consensus results. The graphical output results included heatmaps of the consensus matrices, which displayed the clustering results, consensus cumulative distribution function (CDF) plot and delta area plot, which allowed us to determine an approximate number of clusters. The criteria to determine the number of clusters we considered, were that the consistency within the cluster was relatively high, the coefficient of variation was relatively low, and there was no appreciable increase in the area under the CDF curve. The coefficient of variation was calculated according to the following formula: CV = (SD/MN)*100%, where SD represents the standard deviation and MN represents the average of samples. The category number was selected as the area under the CDF curve and showed no significant change. In fact, for the purpose of fine classification of breast cancer, we preferred to choose a larger number of categories. The heatmap corresponding to the consensus clustering was generated by pheatmap R package.

### 2.5 Survival and clinical characteristics analyses

Kaplan–Meier plots were used to illustrate overall survival among breast cancer subgroups defined by DNA methylation profiles. The log‐rank test was used to evaluate the significance differences among the clusters. Survival analyses were performed using the survival package in R. Associations between clinical and biological characteristics with DNA methylation clustering were analysed using the chi‐square test. All tests were two‐sided and for all statistical tests, *P* < 0.05 was considered significant unless otherwise noted.

### 2.6 Specific DNA methylation markers for breast cancer subgroups

In this analysis, a quantitative approach for quantitative differentially methylated regions (QDMRs), previously developed to quantify methylation differences and identify DMRs from genome‐wide methylation profiles by adapting Shannon entropy (Zhang *et al*., 2011), was used to find the specific DNA methylation CpGs that were specifically hypermethylated or hypomethylated within particular breast tumour subgroups as described above. The quantification of DNA methylation difference across large numbers of samples and the identification of sample specificity play important roles in genomic functional analyses. DMRs with different methylation status among multiple samples were regarded as possible epigenetic functional regions involved in transcriptional regulation. Thus, the identification of DMRs among multiple samples provided a more comprehensive survey for this study. With the rapid development of high‐throughput detection technology, there have been considerable efforts made to identify DMRs from methylation profiles. However, the development of DNA methylation measurements poses significant challenges for concurrent DMR methods. Shannon entropy, a quantitative measure of differences and uncertainty in datasets, has been widely applied in quantitative biology, such as identifying potential drug targets and tissue‐specific genes. To quantify methylation differences and further identify DMRs across multiple samples, Zhang *et al*. (2011) adapted the Shannon entropy model and developed an improved approach, termed the quantitative differentially methylated region (QDMR). QDMR is an effective tool for quantifying methylation differences and identifying DMRs across multiple samples. This approach can give a reasonable quantitative measure of methylation differences across multiple samples as well. We used the threshold that was determined by QDMR from the methylation probability model. QDMR can also measure the sample specificity of each DMR. For each DMR *r,* the entropy *H*_Q_ represents the methylation difference across all samples. For each sample *S*, the entropy $H_{Q/\bar{S}}$ is the difference across samples that do not include sample *S*. Thus, the contribution of sample *S* to the whole methylation difference can be reflected by the entropy difference as:

(3) $$\Delta H_{r/S}=H_{Q/\bar{S}}-H_{Q}$$

The categorical sample‐specificity *CS*_*r/s*_ can be defined as:

(4) $$CS_{r/S}=\{\begin{array}{cc} & \Delta H_{r/S}\times {\text{sign}}_{r,S},\Delta H_{r,S}>0\\ & 0,\;\Delta H_{r/S}\leq 0 \end{array}\}$$

where sign_*r,s*_ is the sign of the difference between methylation level *m*_*r/s*_ in sample *S* and the median methylation level of vector *m*_*r*_ in region *r*, as described by Zhang *et al*. (2011). Thus, the subgroup with the maximal absolute of the categorical sample‐specificity *CS*_*r/s*_ was determined as the specific subgroup corresponding to the particular CpG site.

### 2.7 Constructing the prognosis model based on Bayesian network classification and the model test

To validate specific CpG sites, a supervised Bayesian network classification model was constructed using the train set. The samples in the test set were assigned to the corresponding subgroups using this classification model. An additional external validation dataset from Gene Expression Omnibus (GEO, accession number GSE72251; Edgar *et al*., 2002), which included 119 breast cancer samples, was also included in the test of the prognosis model. All of the samples in the external validation dataset were assigned a class label using the prognosis model like the test set in TCGA. The performance of the model was evaluated using the accuracy rate. The receiver operating characteristic curve (ROC) was obtained using the pROC package in the R programme.

### 2.8 Functional classification of gene sets

g: Profiler (Reimand *et al*., 2016), a web server for functional interpretation of gene lists, was used to perform gene enrichment analysis of Gene Ontology, Biological pathways, Regulatory motifs in DNA and Protein databases of genes.

## 3 Results

### 3.1 DNA methylation features for classification based on prognosis

The DNA methylation profiles of breast cancers from the TCGA database were used to cluster breast cancer prognostic molecular subgroups. First, a series of pre‐processing steps were performed, including adapting missing values, removing batch effects, removing sex chromosomes and single nucleotide polymorphisms, and extracting CpGs in promoter regions (Section 2). The samples were then separated into two cohorts: the train and test sets (Section 2). For each of the CpG sites in the train set (which contained 335 tumour samples), a univariate COX proportional risk regression model was constructed with methylation levels of the CpGs and survival information of cases. Through this analysis, 6760 CpG sites were significant (*P *< 0.05), i.e. influenced patient survival. Age (*P* = 0.0052) and stage (*P *= 0.0008) were also significant factors. Next, the significant CpGs were introduced into multivariate COX proportional risk regression models using age and stage as covariates to find independent prognostic factors. Ultimately, 3869 CpG sites were significant and were used as the final classification features (Table S1).

### 3.2 Consensus clustering of breast tumours identified distinct DNA methylation prognosis subgroups

**Figure 1:**
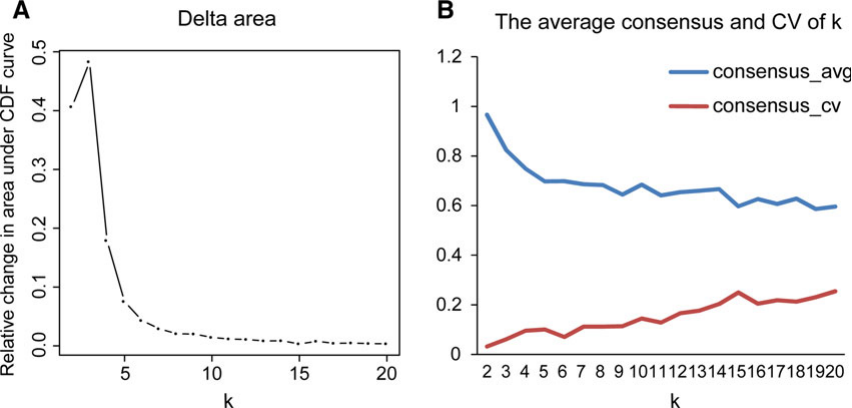
Criteria for the selection of the number of categories. (A) Delta area curve of consensus clustering, indicating the relative change in area under the cumulative distribution function (CDF) curve for each category number *k* compared with *k* – 1. The horizontal axis represents the category number *k* and the vertical axis represents the relative change in area under CDF curve. (B) The average cluster consensus and coefficient of variation among clusters for each category number *k*. The blue line represents the average cluster consensus and the red line represents the coefficient of variation among clusters.

**Figure 2:**
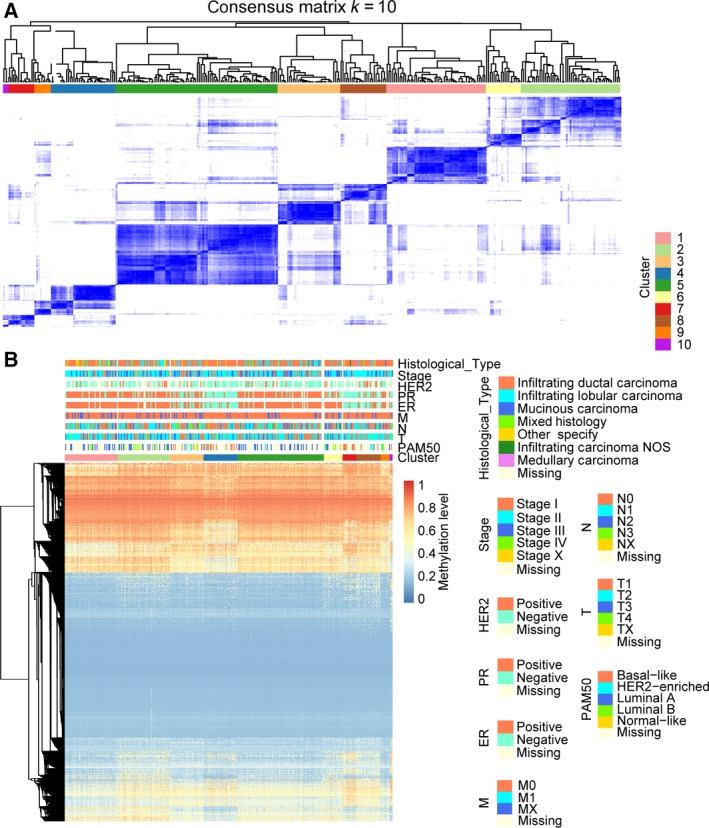
Consensus matrix for DNA methylation classification with the corresponding heat map. (A) The colour‐coded heatmap corresponding to the consensus matrix for *k* = 10 obtained by applying consensus clustering. The colour gradients were from 0 to 1, representing the degree of consensus, with white corresponding to 0 and dark blue to 1. (B) The heatmap corresponding to the dendrogram in (A) which was generated using the pheatmap function with DNA methylation classification, PAM50 classification, estrogen receptor, progesterone receptor, HER2 receptor status, TNM stage, clinicopathological stage and histological type as the annotations.

Next, consensus clustering based on the β‐values of the 3869 independent prognosis‐associated CpG sites was performed to obtain distinct DNA methylation prognostic molecular subtypes of breast cancer. To determine the appropriate number of classes, the average cluster consensus and the coefficient of variation among clusters were calculated for each category number. The area under the CDF curve began to stabilize after 10 categories (Fig. 1A). As can be seen from the average cluster consensus curve, 10 categories led the curve to an upward inflection point (Fig. 1B) and the coefficient of variation of 10 categories was within an acceptable range (Fig. 1B). Therefore, we intended to use 10 as the appropriate number of categories for further analysis. Additionally, the consensus matrix was naturally a better visualization tool to help assess the clustering’ composition and number. We associated a colour gradient of 0–1, with white corresponding to 0 and dark blue corresponding to 1, and assumed that the matrix is arranged so that items belonging to the same cluster are adjacent to each other. In this arrangement, a matrix corresponding to a perfect consensus will show a colour‐coded heatmap characterized by blue blocks along the diagonal on a white background. The colour‐coded heatmap corresponding to the consensus matrix obtained by applying consensus clustering to these cases is shown in Fig. 2A, and represents the consensus for *k* = 10, which displays a well‐defined 10‐block structure. The heatmap corresponding to the dendrogram was generated using the pheatmap function with DNA methylation classification, PAM50 classification, estrogen receptor, progesterone receptor, HER2 receptor status, TNM stages, clinicopathological stages and histological type as the annotations (Fig. 2B). Due to the small sample size of cluster 10 (only three samples included), we regarded these as outliers and did not consider them in the subsequent analysis.

**Figure 3:**
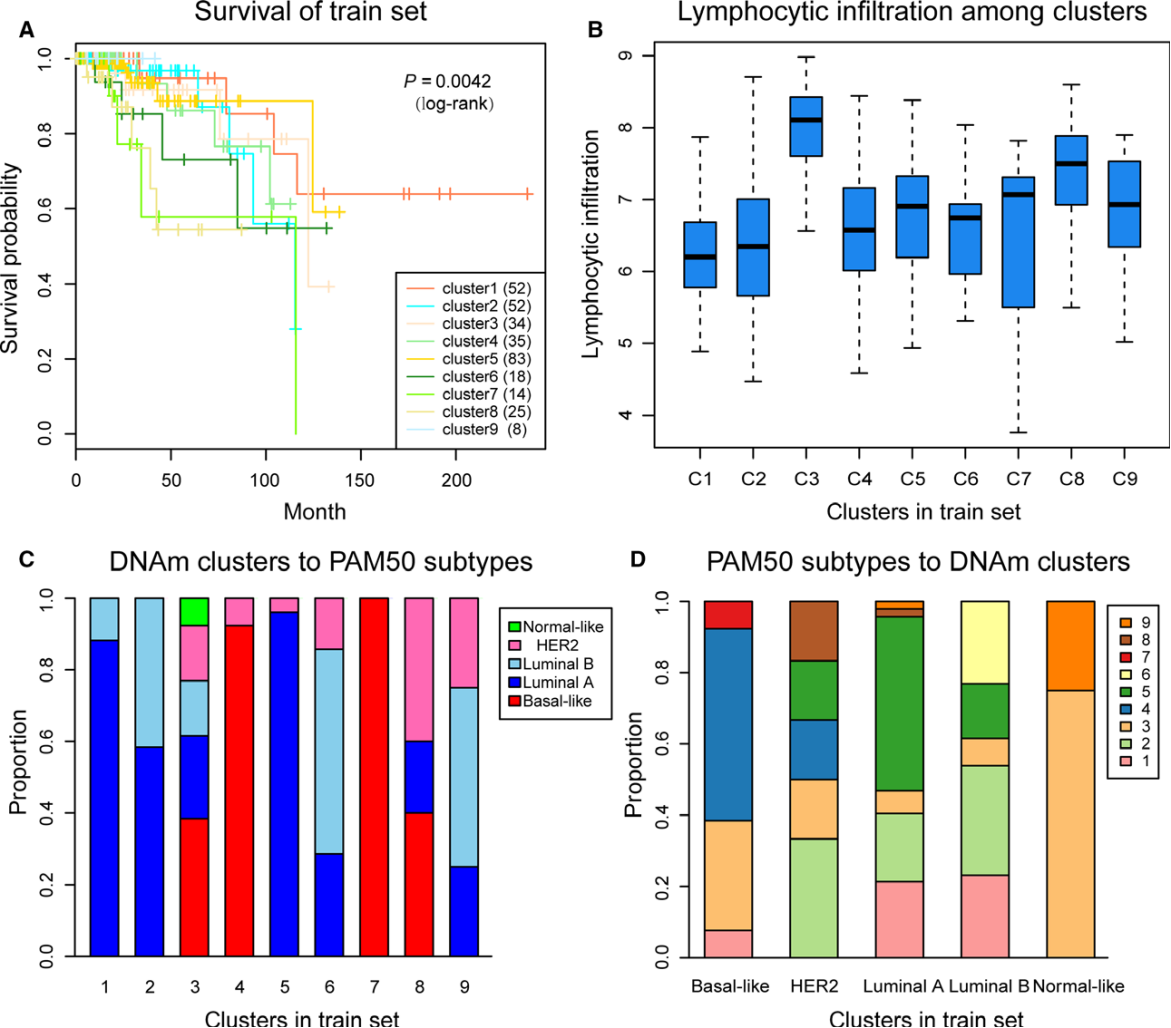
Survival curves of DNA methylation subtypes and the comparison of lymphocyte infiltration between DNA methylation clusters and their PAM50 classifications. (A) The survival curves of DNA methylation subtypes in train set. The horizontal axis represents the survival time (months), and the vertical axis the probability of survival. The numbers in parentheses in the legend represent the number of samples in each cluster . The log‐rank test was used to assess the statistical significance of the difference. (B) Lymphocyte infiltration score distributions of nine DNA methylation clusters in the train set. The horizontal axis represents the DNA methylation clustering. (C) PAM50 subtypes with enrichment in each DNA methylation cluster. (D) The reverse orientation of (C).

Next, we compared the prognosis differences among the remaining nine clusters. Kaplan–Meier survival analysis showed that the outcome differences among these clusters were significant (Fig. 3A). We also performed a log‐rank test between each pair of subgroups and found that the differential prognoses between clusters 2 and 7, clusters 4 and 7, clusters 6 and 7, clusters 2 and 8, clusters 3 and 8, clusters 4 and 8, clusters 5 and 8, and clusters 6 and 8 were all significant.

The PAM50 subtypes, also known as ‘intrinsic’ subtypes of breast cancer (Basal‐like, HER2‐enriched, Luminal A, Luminal B and Normal Breast‐like), have been identified and intensively studied (Perou *et al*., 2000). These groups show critical differences in incidence (Carey *et al*., 2006), survival (Cheang *et al*., 2008) and response to treatment (Prat *et al*., 2010). Some of the DNA methylation subgroups (e.g. clusters 3, 8 and 9) reflected different PAM50 (Cancer Genome Atlas, 2012) subtypes (Fig. 3C), which indicated that patients in different PAM50 subtypes share the same DNA methylation characteristics. Hence, DNA methylation can serve as a commendable biomarker for breast cancer classification. Conversely, different DNA methylation characteristics were also found in the same PAM50 subtype (Fig. 3D). For example, clusters 4 and 7, which have different DNA methylation profiles, were two distinct subgroups of the Basal‐like subtype; and clusters 1 and 5 contained the highest proportion of Luminal A subtype. These results indicated that DNA methylation status represents a more elaborate classification analysis for breast cancers. This is a more detailed explanation of the heterogeneity of breast cancer. Additionally, sometimes the same DNA methylation cluster was classified into different subtypes (e.g. cluster 2 was classified into Luminal A and Luminal B). This might explain the reason for the failure of some therapeutic options, as different therapeutic options are considered for different subtypes, although they may have the same underlying aetiology, such as DNA methylation abnormalities.

### 3.3 Characterizing different characteristics of DNA methylation clustering

Next, we wanted to test whether our DNA methylation subgroups of breast tumours subdivided cases more accurately than the PAM50 classification. In other words, we wanted to estimate whether our DNA methylation clustering corresponding to the same PAM50 subtype had different characteristics including methylation level, pathological parameters and clinical outcome. First, we analysed the DNA methylation clustering which represented enrichment in the same PAM50 subtype.

The methylation levels of the Basal‐like subgroups, clusters 4 and 7, were clearly distinct (Fig. 2B). Moreover, the 5‐year survival rate of the two clusters was significantly different (Fig. S1A). This suggested that patients in the same PAM50 subtype had difference prognoses, albeit that all their prognoses were poor, consistent with previous studies that showed that a poor prognosis for the Basal‐like subtype. We conducted the same survival analyses for Luminal A subgroups (clusters 1, 2 and 5) and Luminal B subgroups (clusters 2 and 6); no significant difference between them was found. For the Luminal A subgroups, clusters 1, 2 and 5, we found that the histological types were significantly different between the three subgroups (*P* = 0.015, chi‐square test), though they did not have any significant difference in prognosis. For the Luminal B subgroups, race (*P* = 0.037), N stage (*P* = 0.005), pathological stage (*P* = 0.036), progesterone receptor status (*P* = 0.026) and presence of metastases (*P* = 0.028) were significantly different between cluster 2 and cluster 6.

The chi‐square test was also used to analyse globally the associations between clinical and biological characteristics with DNA methylation clustering (Table 1). The results showed that race, age, M stage, N stage, pathological stage, ER status, PR status, HER2 status, histological type, and whether there was a distant metastasis were significantly different among the DNA methylation prognosis clusters (*P* < 0.05). This indicated that the heterogeneity of our DNA methylation prognosis clustering could be explained by clinical and biological characteristics.

**Table 1:** The results of chi‐square test on the global level

Clinical attributes	Subclasses	*P*‐value^a^
Age	Young	0.0433
	Old	
Race	White	0.0054
	Asian	
	Black or African American	
	American Indian or Alaska Native	
N stage	N0	0.0045
	N1	
	N2	
	N3	
M stage	M0	0.0173
	M1	
Stage	Stage I	0.0009
	Stage II	
	Stage III	
	Stage IV	
ER	Negative	1.0232e‐32
	Positive	
PR	Negative	2.0291e‐25
	Positive	
HER2	Negative	0.0103
	Positive	
Histological type	Infiltrating ductal carcinoma	0.0003
	Infiltrating lobular carcinoma	
	Medullary carcinoma	
	Mucinous carcinoma	
	Infiltrating carcinoma NOS	
Metastatic	Yes	0.0117
	No	

a *P*‐value: the *P*‐value of chi‐square test.

Lymphocytic infiltration is associated with a better prognosis in breast cancer. Quigley *et al*. (2015) demonstrated that an elevated CTL expression signature, which was used as a surrogate of lymphocytic infiltration, was associated with longer survival in Basal‐like tumours. Next, we explored the lymphocytic infiltration of our classification results using TCGA, with the average expression of CTL used to represent lymphocyte infiltration, as described by Quigley *et al*. The list of CTL genes (103 genes) used was generated using Nanodissect (http://nano.princeton.edu/) in the study of Ju *et al*. (2013). By calculating the lymphocyte infiltration score for each sample of the train set, we found that the overall infiltration of clusters 4 and 7 was lower in the Basal‐like subgroup than in other groups. The result is consistent with the poor prognosis of Basal‐like subtype in previous studies as well as in ours (Fig. 3B). In two subgroups including clusters 3 and 8, with a higher degree of infiltration, the prognosis of these two groups was also relatively better. It is noteworthy that these two subgroups do not belong to any single PAM50 subtype but rather are a mixture of multiple PAM50 subtypes. This indicating that our DNA methylation molecular typing separated subgroups with a high degree of lymphocyte infiltration from the PAM50 subtypes. Cluster 3, particularly, was the only subgroup which consisted of samples from all five PAM50 subtypes, and it had the highest degree of lymphocyte infiltration. Actually, there was a slight positive correlation between the degree of subtype mixing and lymphocyte infiltration (*r* = 0.71, *P* = 0.032).

### 3.4 Identifying specific DNA methylation markers and analysing DNA methylation prognosis subgroups of breast cancer

**Figure 4:**
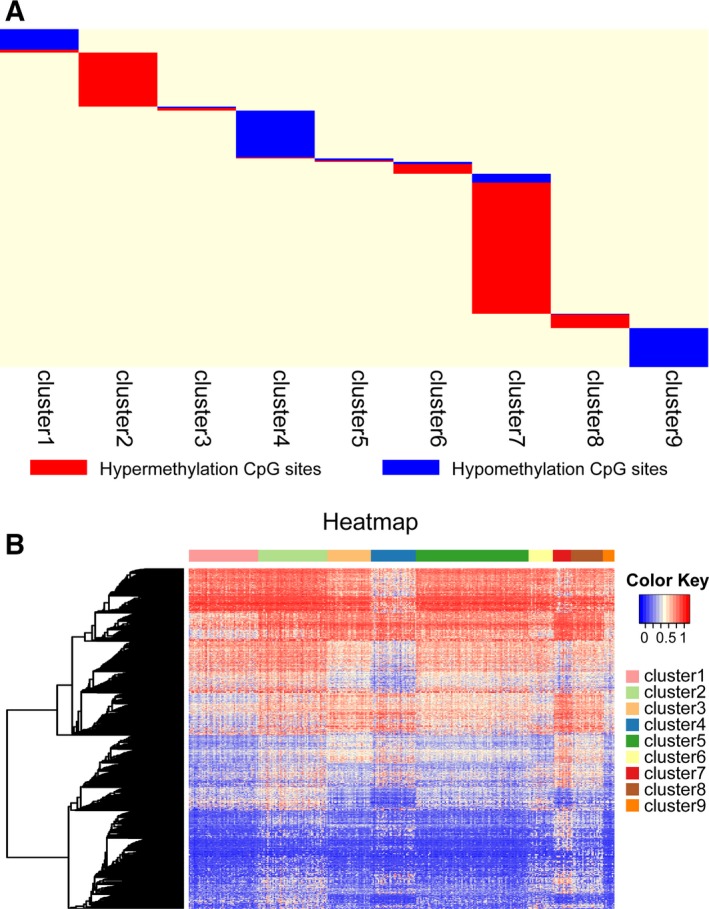
Specific hyper/hypomethylation CpG sites for each DNA methylation cluster. (A) Display of specific CpG sites for each DNA methylation prognosis subtype. The red bars and blue bars represent hypermethylation CpG sites and hypomethylation CpG sites, respectively. (B) The heat map for the specific sites in nine DNA methylation clusters.

To identify the specific hyper/hypomethylation CpG sites that defined particular DNA methylation subgroups of breast cancer, QDMR software developed as a quantitative approach was employed. The 3869 CpG sites across the nine subgroups were used to find specific CpGs in each subgroup. For each subgroup, the mean DNA methylation level of samples for each of the 3869 CpGs was calculated, and a matrix with 3869 × 9 dimensions was input to QDMR. To find the specific CpGs for every subgroup, we lowered the threshold of the SD parameter to 0.04. Finally, 1252 specific hyper/hypomethylation CpG sites, corresponding to 888 genes, were identified (Table S2). These were DNA methylation markers for the different subgroups in breast cancer. The results showed that the number of specific CpGs within each subgroup ranged from 13 to 519 (Fig. 4A,B, Table 2); cluster 7 had the largest number of specific hypermethylated CpGs.

**Table 2:** The numbers of specific CpGs for clustering

Cluster	Number of specific CpGs
Cluster 1	87
Cluster 2	200
Cluster 3	15
Cluster 4	177
Cluster 5	13
Cluster 6	44
Cluster 7	519
Cluster 8	53
Cluster 9	144

Additionally, we explored gene expression within the particular DNA methylation subgroups. We obtained expression values in 332 samples in the train set for 855 of the 888 genes (some of these genes were not detected) from the expression dataset. Among these genes, 285 genes (approximately one‐third) had an average expression value within a particular subgroup that was the maximum or minimum of that in the other subgroups. This strict standard indicated that the specificities of these genes were consistent as to DNA methylation level and gene expression level.

Moreover, we conducted a functional enrichment analysis for the genes corresponding to the specific CpGs for each of DNA methylation clusters, respectively. The top significant terms in each category for each cluster are shown in Fig. S2. The genes of clusters 3, 5, 6 and 8 were collected into a smaller number of terms due to the small gene numbers. As the results showed, specific genes of clusters 1 and 7 were mainly involved in glucuronidation and metabolic process, the genes of cluster 2 were mainly involved in developmental and differentiation processes, the genes of cluster 4 were involved in cortisol metabolic and biosynthetic processes, and the genes of cluster 9 were mainly involved in cell adhesion and signaling. This implies that our different DNA methylation subgroups were involved in different functions and biological pathways.

### 3.5 Constructing and evaluating the prognosis prediction model

**Figure 5:**
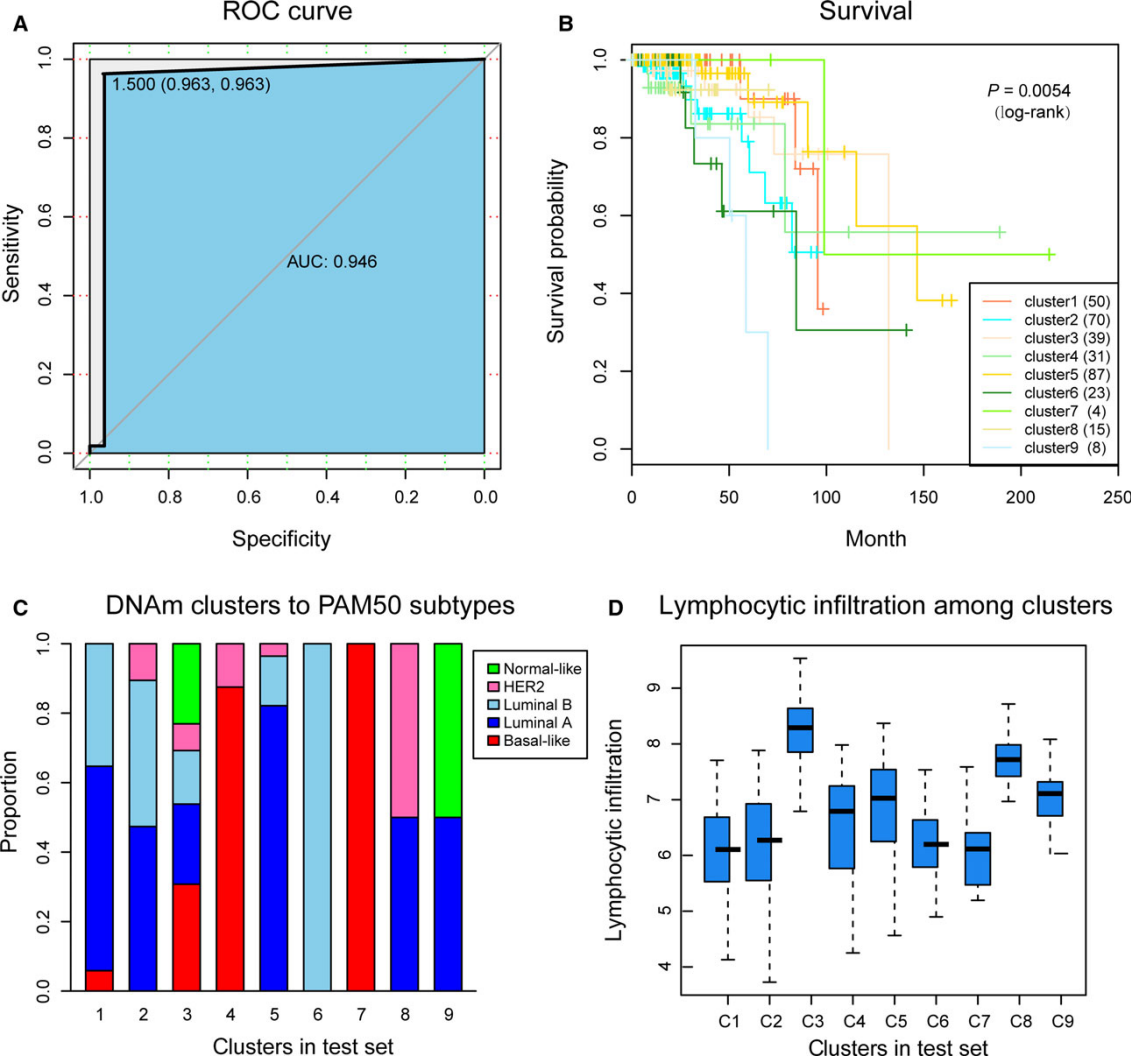
The prognosis model and prediction results. (A) The ROC curve displayed the sensitivity and specificity of the prognosis model. The area under the curve (AUC) reached 0.946. (B) Survival curves of nine clusters predicted from the test set using the prognosis model. The numbers in parentheses in the legend represent the number of samples in each cluster. The log‐rank test was used to assess the statistical significance of the difference. (C) PAM50 subtypes with enrichment in each DNA methylation cluster. (D) Lymphocyte infiltration score distributions of DNA methylation clusters in the test set.

To confirm the discriminatory ability of the specific CpGs for each subgroup obtained by us, a Bayesian network classification was constructed using the train set, with the 1252 specific CpGs used as features. The 10‐fold cross‐validation method was used to evaluate the performance of the model, which obtained a classification accuracy of 90.96% (Table 3). The area under receiver operating characteristic curve reached 0.946 (Fig. 5A).

**Table 3:** The confusion matrix of Bayesian network classification. Each row of the matrix represents the instances in a predicted cluster, and each column represents the instances in an actual cluster. C1–C9 are the logograms for clusters 1–9

	C1	C2	C3	C4	C5	C6	C7	C8	C9
C1	**52**	0	0	0	2	0	0	0	0
C2	2	**51**	0	0	0	0	0	1	0
C3	0	0	**26**	3	3	0	1	1	0
C4	0	0	0	**33**	0	0	1	0	1
C5	2	4	2	0	**78**	0	0	2	0
C6	1	1	0	0	0	**17**	0	0	0
C7	0	0	0	0	0	0	**13**	1	0
C8	0	0	0	0	0	1	0	**24**	0
C9	0	0	0	0	0	0	0	1	**8**

The bold text in the table represent the numbers of instances in each class that have the same prediction cluster and actual cluster.

Next, we employed this prognostic model to predict cases in the test set. The samples in the test set were assigned a class label corresponding to the train set. Survival analysis of the nine clusters in the test set showed that they were significantly prognostically different (Fig. 5B). This indicated that the specific CpGs in this study could be used as prognostic biomarkers for breast cancer. Furthermore, we compared our classification test with the PAM50 subtypes, and explored the degree of lymphocyte infiltration. We used the same method as the train set and obtained consistent results (Fig. 5C). In particular, clusters 4 and 7, as predicted in the test set, still belonged to the Basal‐like subtype, whereas clusters 1, 2 and 5 were subgroups of the Luminal A subtype, and clusters 2 and 6 were subgroups of the Luminal B subtype. The degree of lymphocyte infiltration of the clustering in the test set was also consistent with the train set (Fig. 5D). These results further illustrate the predictive accuracy of our model and the stability of its features. In addition, we conducted survival analyses for the PAM50 subgroups and obtained consistent results for Luminal A and Luminal B subgroups. However, in the Basal‐like subgroup, clusters 4 and 7 were no longer significant, which may be due to the very small sample sizes in cluster 7 (only four samples were predicted for cluster 7).

Because the limitation of the survival data in the TCGA for breast cancer may reduce the merit of our results, we added an additional external validation dataset from the Gene Expression Omnibus (GEO, accession number GSE72251), which included 119 breast cancer samples. As in the TCGA database test set, all samples in the external validation dataset were assigned a class label using the prognosis model constructed from the train TCGA dataset. Survival analysis of the nine clusters was then performed. The result showed that the prognoses among them were significantly different (*P* = 0.0188, Fig. S3). We also explored the survival between the PAM50 subgroups and obtained results consistent with the TCGA test set. This indicated the portability of our prognosis prediction model. To check whether prognoses of clustering in test set and the external validation dataset were similar to the corresponding clustering in the train set, we compared the same labelled clusters in the train and test sets or external validation dataset, respectively; there was no significant difference between any of the groups (Figs S4 and S5). These results show that cases that were classified or predicted to be in the same DNA methylation subgroups, had the same prognosis.

## 4 Discussion

Recent developments in sequencing technologies have made it possible to analyse genome‐wide DNA methylation profiles at high resolution. Whole genome bisulfite sequencing is the best method to investigate DNA methylation; however, its efficacy is limited by high cost and analytical burden. DNA methylation arrays are a good alternative for investigating genome‐wide DNA methylation in a large collection of tumours. The TCGA database is a publicly available resource covering a wide variety of data types in a variety of cancers. The Infinium HumanMethylation450 BeadChip array dataset of breast cancer contains a large number of samples that were downloaded from TCGA for our classification analysis. The large sample sizes allowed us to explore the molecular subtypes of breast cancer more comprehensively.

Precision medicine in cancer treatment is based on the assumption that every patient has a unique variation of genetic alterations and should be treated accordingly. Thus, for personalized medicine to be effective, it is necessary to achieve a detailed classification of the cancer genome and epigenome. Many studies have suggested that epigenetic modifications (DNA methylation) play a pivotal role in early detection, improved molecular classification, prognosis and adjuvant treatment of breast cancer. These opinions suggest that the level of analysis could have important biological and clinical implications in the era of precision medicine (Hu and Zhou, 2017; Pasculli *et al*., 2018). Moreover, classifications based solely on the tissue of origin or pathological features have shown limitations. We therefore conducted this study to obtain detailed classifications of the breast cancer epigenome based on DNA methylation. We first selected prognosis‐associated CpG sites within gene promoter regions for cluster analysis. Nine different prognosis subgroups were obtained by consensus clustering, with either molecular or clinical differences among them, which confirmed the heterogeneity of breast tumours and the necessity of meticulous classification.

Consensus clustering provided the recommended number of clusters as well as determining the cluster assignments compared with other unsupervised clustering methods, such as hierarchical clustering. It is noteworthy that the results of consensus clustering were dependent on the inner‐loop clustering of choice (*k*‐means in the experiments), with consensus clustering based on *k*‐means producing slightly better results than others (including HC and SOM). This indicates that every clustering method has its own idiosyncrasies, related to the measure of similarity used to compare and group data items. We first suggest dividing breast tumours into nine prognosis‐subgroups on the basis of DNA methylation. This level of detail brings about a high intra‐class consistency to better guide personalized medicine.

Among these nine subgroups, we found two Basal‐like subgroups, three Luminal A subgroups and two Luminal B subgroups. The differences at the molecular level, clinical attributes and prognosis indicated that there is still significant heterogeneity within the PAM50 subtypes of breast cancer. A more detailed classification could contribute to the realization of personalized medicine.

In QDMR analysis, we found 1252 specific hyper/hypomethylation CpG sites, corresponding to 888 genes, which defined particular DNA methylation subgroups of breast cancer. These sites can be regarded as targets for precision medicine and biomarkers for diagnosing breast cancer. Moreover, the prognosis model using these specific CpGs as features could distinguish the test and external validation datasets into different prognosis clusters that were consistent with the classification results. Many of these specific CpG sites have previously been reported to be associated with breast cancer. Among them, the ketone body production enzyme BDH1 has been proved to be preferentially expressed in the stroma of human breast cancer samples (Martinez‐Outschoorn *et al*., 2012). ABCA1 expression was shown to be regulated by miR‐96 in breast cancer cell lines (Moazzeni *et al*., 2017) and AGTR1 was suggested to be a marker of resistance to neoadjuvant chemotherapy in HER2^−^ breast cancer (de Ronde *et al*., 2013), whereas it was shown to be a therapeutic target in ER^+^ and ERBB2^−^ breast cases (Ateeq *et al*., 2009). In addition, HRH2 activation in breast cancer cells was suggested to increase tumour proliferation (Cricco *et al*., 1994; Davio *et al*., 1994), whereas blocking HRH2 was reported to improve disease‐free survival in breast cancer patients (Parshad *et al*., 2005). Tumour cell MMP3 expression was reported to be a prognostic factor for poor survival in breast cancer (Mehner *et al*., 2015), and MAP2 expression was shown to be significantly associated with pathological responses to neoadjuvant chemotherapy, regardless of breast cancer subtype (Kolacinska *et al*., 2012). Eterno *et al*. (2014) suggested that expression of the HGF receptor c‐Met could be predictive of recurrence after autologous fat graft in post‐surgery breast cancer patients.

## 5 Conclusions

Our research identified nine different prognosis‐subgroups using the data of breast tumours in TCGA that were different at either the molecular level or in epidemiology. This gives a more detailed explanation of the heterogeneity of breast cancer. The specific CpG sites and genes for particular subgroups can serve as biomarkers for personalized treatments. Changes in DNA methylation (hypo/hypermethylation) can be used as markers to diagnose particular subgroups, and clinicians can develop personalized treatments according to these prognoses. Our methods can also be used to study other tumours.

## Author contributions

YZ and DZ: conceived and designed the experiments. JZ, CC and BZ: collected the data. SZ, YW and YG: performed the analysis. YW, ZG and HL: participated in the discussion of the algorithm. SZ and YH: prepared and edited the manuscript. All authors have read and approved the final manuscript.

## Supporting information


**Fig. S1.** The survival curves of DNA methylation subgroups in three PAM50 subtypes.
**Fig. S2.** The enrichment analyses of genes corresponding to the specific CpG sites in each DNA methylation cluster.
**Fig. S3.** The survival curve of nine predicted subgroups in the external validation dataset.
**Fig. S4.** The survival curves of the same labelled clusters in the train set and test set.
**Fig. S5.** The survival curves of the same labelled clusters in the train set and external validation dataset.

**Table S1.** Classification features.

**Table S2.** The specific hyper/hypomethylation CpG sites and the corresponding genes.
