# Supplementary material for: Specific breast cancer prognosis‐subtype distinctions based on DNA methylation patterns
Source: Mol Oncol. 2018 May 21;12(7):1047–60. doi: 10.1002/1878-0261.12309 (PMC6026876; doi:10.1002/1878-0261.12309)
Supplement: Supplementary file 1 — Fig. S1. The survival curves of DNA methylation subgroups in three PAM50 subtypes. Fig. S2. The enrichment analyses of genes corresponding to the specific CpG sites in each DNA methylation cluster. Fig. S3. The survival curve of nine predicted subgroups in the external validation dataset. Fig. S4. The survival curves of the same labelled clusters in the train set and test set. Fig. S5. The survival curves of the same labelled clusters in the train set and external validation dataset. [file MOL2-12-1047-s001.pdf]

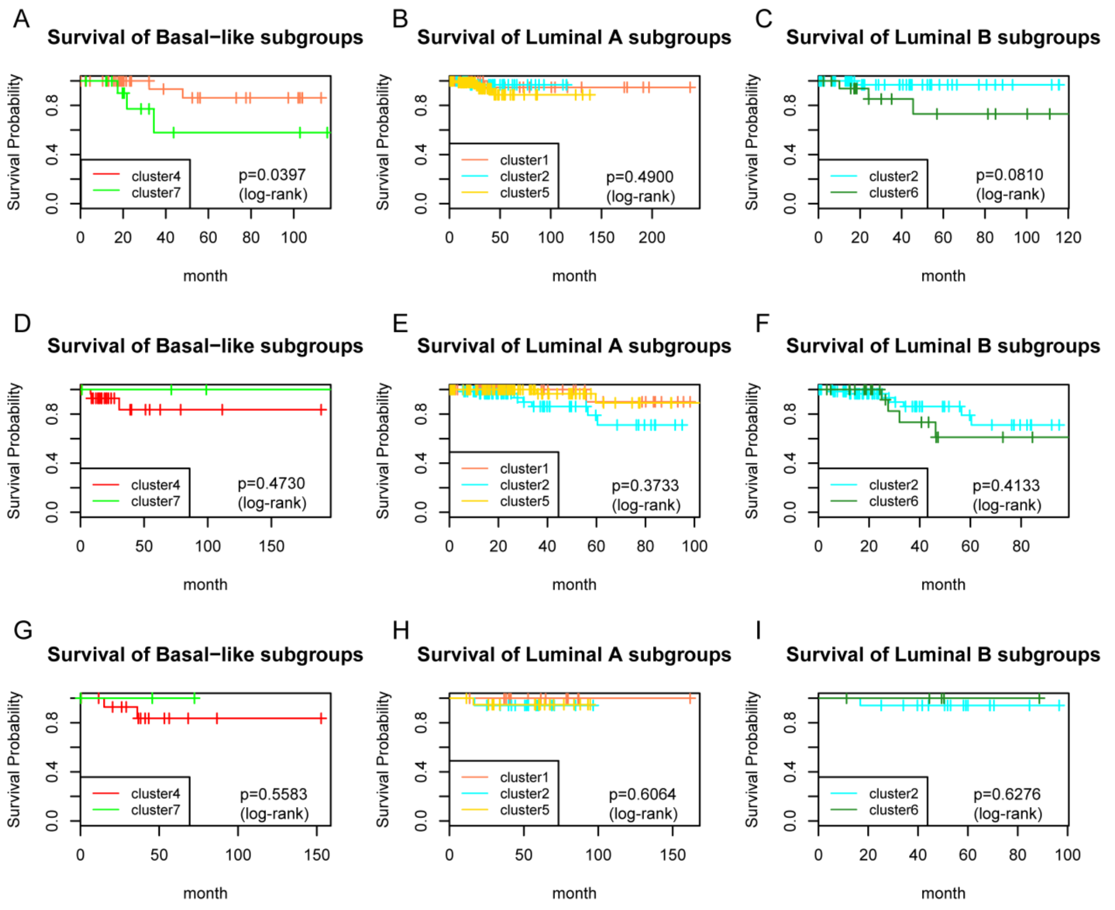

Supplementary Figure S1. The survival curves of DNA methylation subgroups in three PAM50 subtypes. A. The survival curve of two Basal-like subgroups in train set. B. The survival curve of three Luminal A subgroups in train set. C. The survival curve of two Luminal B subgroups in train set. D. The survival curve of two Basal-like subgroups in test set. E. The survival curve of three Luminal A subgroups in test set. F. The survival curve of two Luminal B subgroups in test set. G. The survival curve of two Basal-like subgroups in external validation dataset. H. The survival curve of three Luminal A subgroups in external validation dataset. I. The survival curve of two Luminal B subgroups in external validation dataset. The log-rank test was used to assess the statistical significance of the difference.

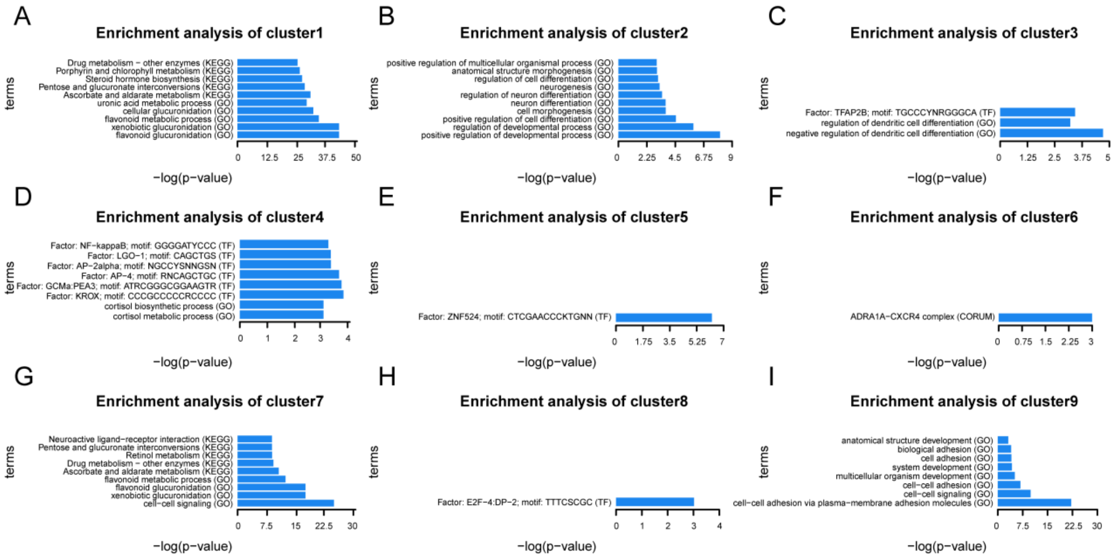

Supplementary Figure S2. The enrichment analyses of genes corresponding to the specific CpG sites in each DNA methylation cluster.

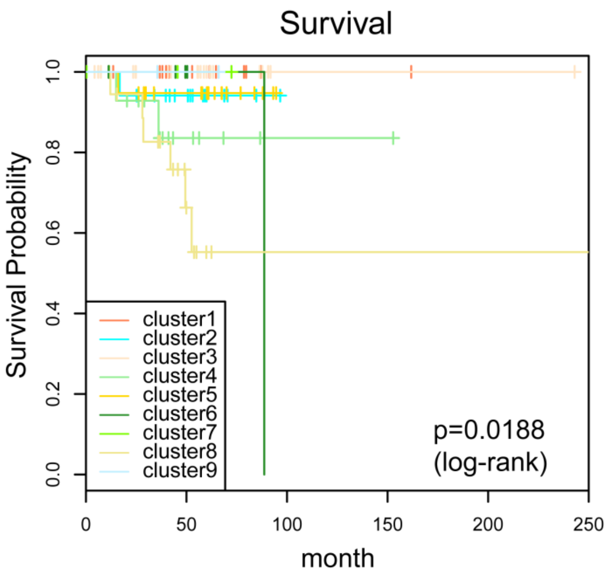

Supplementary Figure S3. The survival curve of nine predicted subgroups in the external validation dataset. The log-rank test was used to assess the statistical significance of the difference.

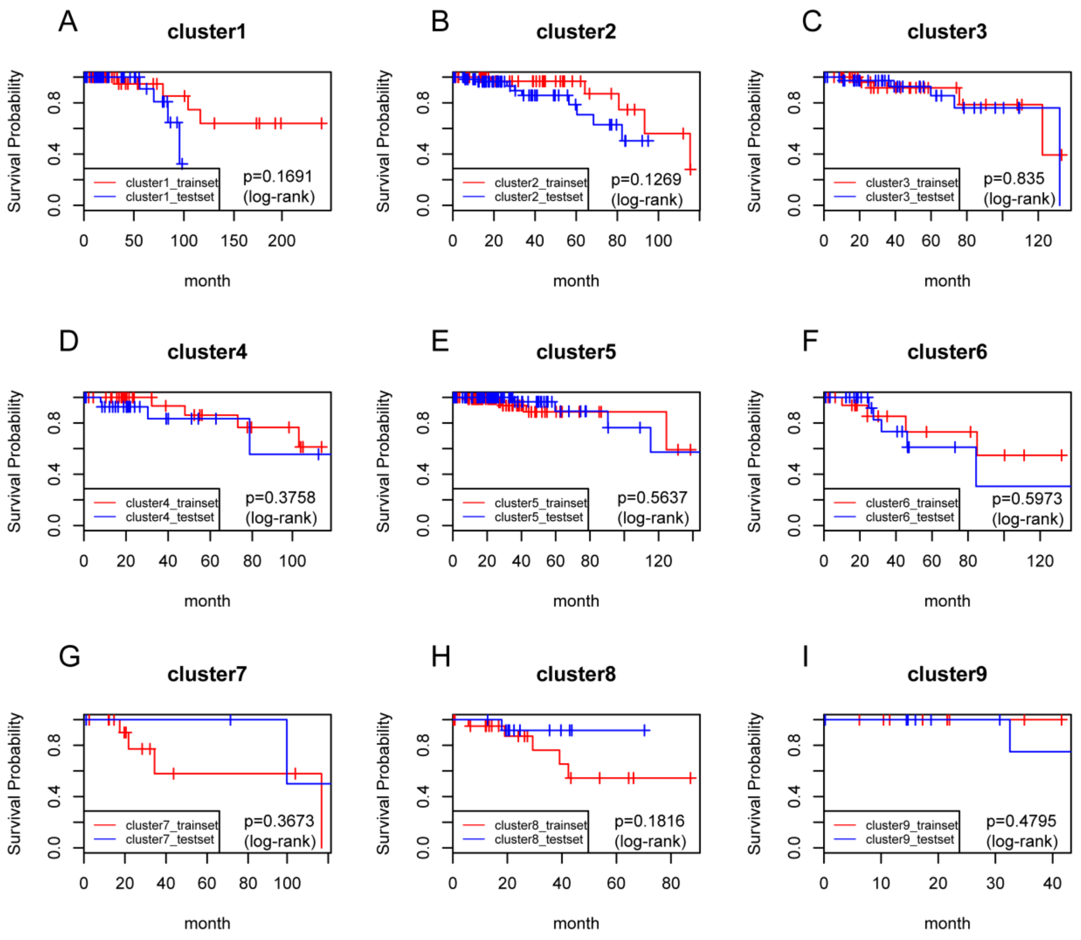

Supplementary Figure S4. The survival curves of the same labeled clusters in train set and test set. The log-rank test was used to assess the statistical significance of the difference.

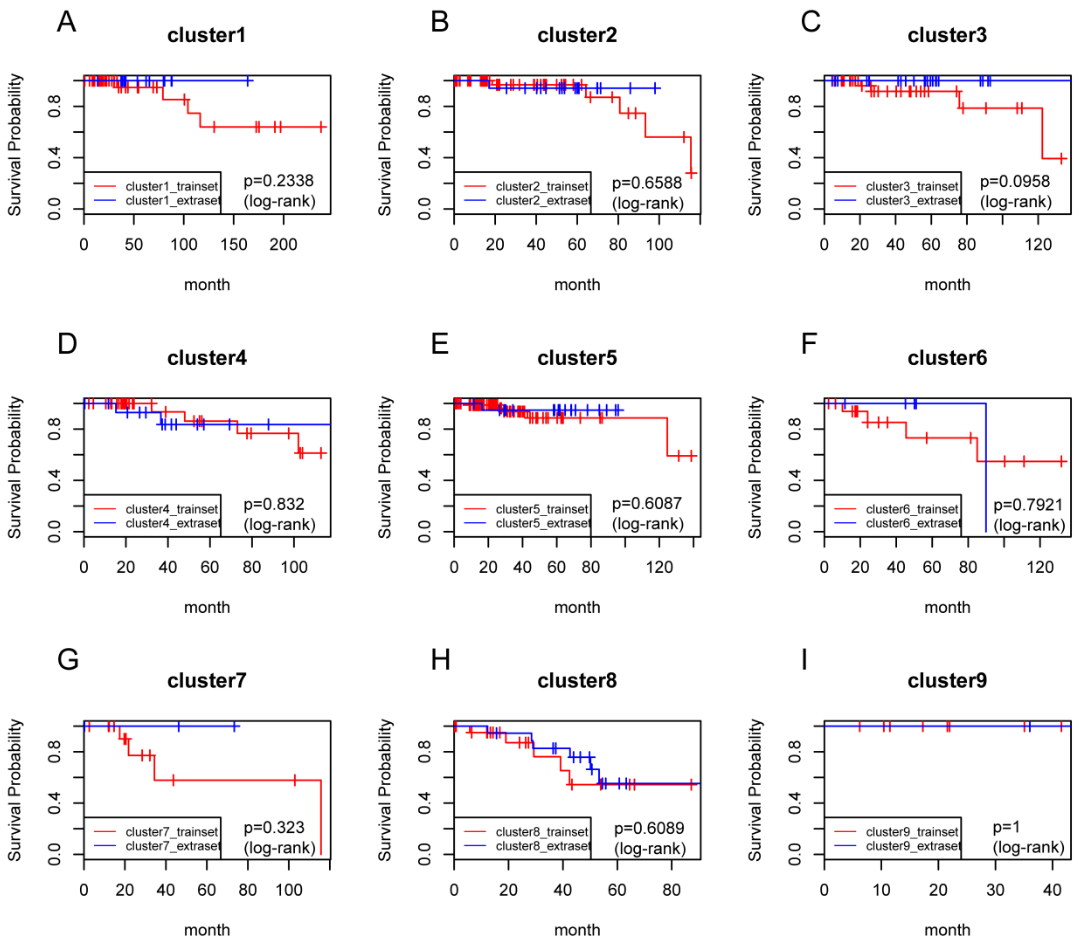

Supplementary Figure S5. The survival curves of the same labeled clusters in train set and external validation dataset. The log-rank test was used to assess the statistical significance of the difference.
